# Exploring motivations and barriers in prostate cancer screening: lessons from a volunteer-based MRI screening study

**DOI:** 10.3389/fpubh.2025.1646494

**Published:** 2025-11-17

**Authors:** Miroslav Světlák, Michal Standara, Tatiana Malatincová, Michal Staník, David Miklánek, Kateřina Hejcmanová, Miloš Pacal, Roman Hrabec, Ondřej Ngo, Karel Hejduk, Jan Křístek, Michal Uher, Ondřej Májek, Alexandr Poprach

**Affiliations:** 1 Faculty of Medicine, Department of Medical Psychology and Ethics, Masaryk University, Brno, Czechia; 2 Department of Radiology, Masaryk Memorial Cancer Institute, Brno, Czechia; 3 Department of Medical Imaging, Faculty of Medicine, Masaryk University, Brno, Czechia; 4 Faculty of Medicine, Department of Urologic Oncology, Masaryk Memorial Cancer Institute, Masaryk University, Brno, Czechia; 4 Faculty of Medicine, Department of Surgical Oncology, Masaryk Memorial Cancer Institute, Masaryk University, Brno, Czechia; 6 Faculty of Medicine, Institute of Biostatistics and Analyses, Masaryk University, Brno, Czechia; 7 National Screening Centre, Institute of Health Information and Statistics of the Czech Republic, Prague, Czechia; 8 Research Group Bioinformatics, Masaryk Memorial Cancer Institute, Brno, Czechia; 9 Faculty of Medicine, Department of Comprehensive Cancer Care, Masaryk Memorial Cancer Institute, Masaryk University, Brno, Czechia

**Keywords:** prostate cancer screening, PSA testing, biparametric MRI, decisional balance scale, early detection, ProstaPilot study, performance

## Abstract

**Background:**

Prostate cancer remains a significant public health challenge, an early detection with prostate-specific antigen (PSA) testing and biparametric MRI (bpMRI) can improve outcomes. However, participation hinges on motivational, psychological, and logistical factors. This study examines the motivational profile of men in the ProstaPilot study to guide strategies to increase uptake of state-of-the-art prostate cancer screening programs.

**Methods:**

The ProstaPilot study enrolled 423 men who underwent both PSA testing and bpMRI of the prostate. Positive results (PSA ≥ 3 μg/L or PI-RADS 4–5 lesions) were referred for further urological examination and biopsy. Using an exploratory correlational design, 360 participants completed a detailed questionnaire. Motivational factors were extracted via Principal Component Analysis (PCA) with Oblimin rotation. Perceptions of prostate cancer risk, severity, and prevention were rated on 1–10 scales (10 = most positive).

**Results:**

PCA identified four motivational factors explaining 55.6% of variance: (1) concerns about screening (e.g., unnecessary surgery, loss of control); (2) perceived benefits of early detection; (3) social motivation (e.g., contributing to research, role modeling); and (4) barriers (e.g., logistics, embarrassment). Over half (51.1%) had not considered screening before ProstaPilot; others decided over varying timeframes. Participants showed high awareness of prostate cancer and valued early detection, rating screening effectiveness 9.55 ± 0.98 and trust in healthcare professionals 9.6 ± 1.0. Social/familial influences were moderate. Satisfaction was high: likelihood to recommend 9.45 ± 1.22; confidence in continuing participation 9.9 ± 0.39.

**Conclusion:**

Highly motivated participants were marked by strong knowledge of prostate cancer screening, trust in healthcare providers, supportive social context, and high personal commitment. These findings support personalized, socially supportive, educational strategies to increase uptake of state-of-the-art screening.

**Clinical trial registration:**

## 1 Introduction

Prostate cancer (PCa) is the most common cancer among men in Europe, creating an essential demand for effective screening programs to reduce healthcare costs, morbidity, and mortality associated with the disease (1). Recent research highlights the potential cost-effectiveness of risk-based screening approaches that integrate PSA (prostate-specific antigen) and magnetic resonance imaging (MRI), which could significantly enhance health outcomes while managing costs (1). The PSA marker is organ-specific, not tumor-specific, so its use without additional criteria has led to over-diagnosis and over-treatment. Additional imaging examinations using MRI and individualized risk assessment help reduce the number of unnecessary biopsies and, consequently, clinically insignificant carcinomas, the detection of which burdens the patient with unnecessary follow-up or even invasive treatment without threatening their life. However, MRI-based screening is inherently more complex, requiring greater time and effort from participants. In 2024, a nationwide pilot screening program combining PSA testing and selective bpMRI (biparametric MRI) was launched.

Despite advancements in screening methods and its reduced incidence and improved survival (2), a significant challenge remains, how to motivate men to participate actively and consistently in these preventive programs. Research indicates that men’s motivation to undergo screening is often shaped by trusted healthcare providers, social encouragement, and targeted educational efforts (3). Addressing men’s fears, misconceptions, and the invasive nature of traditional screening methods is crucial to improving participation rates.

Prostate cancer screening practices vary widely across regions, with participation rates ranging from as high as 82% in some European studies (4) to significantly lower rates in regions like Kenya and Nigeria, where only 5–28% of men undergo testing (5, 6). In the Czech Republic, opportunistic prostate-specific antigen (PSA) screening is moderately prevalent, reaching 45% in the target age group of 50–69 years (7).

Prostate cancer screening is influenced by a range of individual, social, and systemic factors, with both significant benefits and notable challenges. A primary motivator for men to undergo screening is the perception of risk, particularly among those with a family history of prostate cancer or who receive recommendations from healthcare professionals (3, 8). Social prompting, including encouragement from family or friends and trust in physicians, also plays a significant role (3). Key benefits of screening include the potential for early detection and subsequent reassurance regarding one’s health, which can lead to timely treatment if necessary (9). However, substantial barriers exist, including fear of cancer diagnosis, embarrassment about procedures like digital rectal exams, and skepticism about the necessity or accuracy of PSA tests (8). Additionally, concerns about overdiagnosis and overtreatment—leading to unnecessary interventions and complications such as urinary incontinence or erectile dysfunction—are major drawbacks highlighted in the literature (3). The role of decision aids in addressing these issues has been studied, showing that while they improve knowledge and reduce decisional conflict, they do not significantly increase screening uptake or discussions between patients and physicians (10). Together, these findings underscore the need for informed, shared decision-making that aligns screening decisions with individual values and preferences while addressing the associated risks and challenges comprehensively.

Recently, bpMRI has been introduced for prostate cancer diagnostics, offering significant advantages over multiparametric MRI (mpMRI), including a shorter examination time, less invasiveness, and the omission of contrast agent application. Several studies have shown bpMRI to be non-inferior to mpMRI and have explored its potential as a primary screening test in both general and high-risk populations (11–13). To assess the feasibility and utility of incorporating bpMRI into a screening protocol in the Czech context, the ProstaPilot study required all participants to commit to a rigorous process, including bpMRI, PSA testing, and biopsies where indicated. It has become clear that in order to encourage men to undertake such a comprehensive and demanding screening procedure, it is important for health professionals to have a good understanding of their motivations.

Given the complexities of MRI-based screening and the significant demands placed on participants, we designed an exploratory correlational study to investigate the motivational landscape of men involved in ProstaPilot study. Participants were enrolled in ProstaPilot primarily for screening. The motivation/barrier survey was an ancillary exploratory component and did not affect screening eligibility. By identifying psychological, social, and practical factors influencing their decisions, we aim to provide actionable insights for designing effective strategies to promote participation in state-of-the-art prostate cancer screening programs.

## 2 Subjects and methods

### 2.1 Participants

Between May 2022 and May 2023, 423 volunteer men were enrolled in the ProstaPilot study. Eligibility criteria included a life expectancy of at least 10 years and the ability to undergo all planned procedures, with no contraindications to MRI or prostate biopsy. All participants underwent both a PSA test and bpMRI of the prostate. The tests were considered positive if the PSA level was 3 μg/L or higher, or if a PI-RADS 4–5 lesion was identified on the MRI. If either test was positive, the patients were subsequently referred to a urologist, who performed a digital rectal examination and recommended a prostate biopsy. See details in the ProstaPilot study (14).

Of these, 360 men completed a questionnaire essential for the present exploratory analysis. Informed consent was obtained from all participants, and data were pseudonymized to protect confidentiality. The study was approved by the institutional Ethics Committee (2022/1303/MOU) and conducted in accordance with the STARD (Standards for Reporting Diagnostic Accuracy Studies) guidelines (15).

### 2.2 Participant recruitment

Recruitment was conducted by the institutional Cancer Prevention Center and through collaborating local general practitioners. The initial questionnaire was distributed to participants during their PSA collection at the Cancer Prevention Center. Participants were instructed to bring this completed form to their MRI appointment, which could occur on the same day or up to two weeks later. The participants who forgot to bring the completed forms to the appointment were provided with a replacement form to fill out as they were waiting for their MRI session.

Men with a history of hip replacement or a known BRCA1/BRCA2 mutation were excluded. Participants were required to confirm that they had not undergone a previous prostate biopsy, prostate MRI, or PSA test in the last two years, and that they had not experienced a urinary infection within the previous six months.

### 2.3 Patient motivation questionnaire

The Pros and Cons questionnaire was developed by combining insights from existing literature, our clinical experience, and theoretical frameworks, including the Transtheoretical Model (16). Specifically, we were inspired by the model’s constructs of decisional balance and its pros and cons scales, which weigh perceived benefits and barriers to behavioral change. This framework helped guide the categorization of influences into domains such as concerns, benefits, social motivations, and barriers (17). Key information about barriers, motivations, and decision-making factors related to prostate cancer screening was drawn from analyzed studies (3, 8, 18). Additionally, data on the effectiveness and limitations of screening modalities, such as PSA tests and MRI, were included based on recent clinical research (9). By synthesizing this knowledge with clinical insights, we created a structured tool for assessing patient decision-making in the ProstaPilot project.

The scale consists of 38 items, structured to assess factors influencing decision-making. Items are rated on a 5-point Likert scale, ranging from 1 (“No influence”) to 5 (“Strong influence”), capturing the extent to which various factors impact individual decisions (Table 1).

**Table 1:** Perceived pros and cons of screening among participants.

What influences my decision to join the prostate cancer prevention program and the ProstaPilot project	Item statistics		Factor loadings			
No influence - score 1…. Has an influence - score of 5	*M*	SD	Concerns (F1)	Benefits (F2)	Social motivation (F3)	Barriers (F4)
Fear of unnecessary surgery	1.7	1.2	0.9			
Fear of unnecessary examinations	1.6	1.0	0.79			
Fear of losing control by becoming entangled in the medical system	1.6	1.0	0.75			
Fear that the results may change my life	1.9	1.2	0.72			
Concern that subsequent procedures may harm my health	1.4	0.9	0.69			
Fear that results may not be reliable or definitive	1.4	0.9	0.69			
Concern that one examination will lead to further ones	1.7	1.0	0.67			
Fear of additional follow-up examinations due to initial results	2.1	1.2	0.63			
Anxiety during the wait for results	1.6	1.0	0.63			
Fear of admitting I’m getting older	1.5	0.9	0.51			
Distrust in the reliability of prostate cancer screening	1.3	0.8	0.46			
Fear of the examination results	1.8	1.1	0.44			
Negative experiences of other men that are widely shared	1.4	0.9	0.35			
Early detection increases the chance of a complete cure	4.5	1.2		0.89		
Feeling of control over my health	4.1	1.4		0.87		
Early detection increases the chance of successful treatment	4.4	1.3		0.86		
Assurance that I am healthy	4.2	1.4		0.84		
Avoiding regret from neglecting my health	4.2	1.4		0.81		
Eliminating uncertainty about my health	4.0	1.4		0.69		
Gaining clarity about any symptoms I may have	3.9	1.5		0.61	0.33	
Contributing to medical research	4.0	1.4		0.61	0.35	
Feeling responsible for taking care of my health	3.8	1.5		0.49	0.48	
Saving money for the health insurance system	2.5	1.6			0.69	
Strengthening my relationship and trust with my doctor	2.8	1.6			0.67	
Being a role model for others	2.4	1.5			0.56	
Fear of the procedure itself (pain, discomfort, unpleasantness)	1.6	1.0	0.38			0.31
My doctor is a man	1.1	0.6				0.78
My doctor is a woman	1.2	0.7				0.75
Fear of being labeled a hypochondriac	1.2	0.8				0.64
Fear of admitting difficulties related to masculinity	1.5	1.0				0.5
Distrust of doctors to discuss or address such issues	1.5	1.0				0.49
Embarrassment about being examined in intimate areas	1.6	1.1				0.47
Time constraints	1.6	1.1				0.44

Beyond the pros and cons, the questionnaire included items designed to capture broader motivational and decisional dimensions of participation in the ProstaPilot study. The results are presented according to the questions in the rest of the questionnaire, reflecting other important dimensions of motivation, such as attitudes, knowledge and needs to participate. The questionnaire utilized a combination of multiple-choice options with the possibility of selecting more than one answer and a scale from 1 to 10 to evaluate various factors, as described in the following study results, with 10 representing the most positive connotation.

### 2.4 Statistical analysis

All statistical analyses were performed using SPSS (version 27.0, IBM Corp.) and TIBCO Statistica (version 13). Demographic data, reasons for participation, decision-making timelines, and perceptions of prostate cancer risk and prevention were summarized using descriptive statistics, including means, standard deviations, and medians for continuous data, and percentages for categorical variables. Data obtained from the pros-and-cons items were analyzed using Principal Component Analysis (PCA) with Oblimin rotation to identify underlying factors. The number of components was determined through parallel analysis conducted with the help of SPSS syntax provided by O’Connor (19). Chi-square tests were used to assess differences between groups of participants. Demographic differences in responses were assessed using one-way ANOVA or independent t-tests.

## 3 Results

Of the 423 men who met the inclusion criteria of the study, 360 completed the questionnaire.

(average age 57.2, median 56.2). There was no significant difference between the groups of those who completed the questionnaire and those who did not, either in terms of education [Pearson χ^2^(2) = 1.30, *p* = 0.52] or age [t(459) = 1.40, *p* = 0.16]. The demographic data of the study population are presented in Table 2.

**Table 2:** Demographic summary of research sample (*N* = 360).

Demographic feature	Number	Percentage (%)
Age group, years		
50–54	144	40.0
55–59	113	31.4
60–64	65	18.0
65–69	38	10.6
Marital status		
Married/registered partnership	279.0	77.5
Divorced	41.0	11.4
In a serious relationship	20.0	5.6
Single	13.0	3.6
Widowed	5.0	1.4
Did not respond	2.0	0.6
Children		
Have children	324.0	90.0
No children	21.0	5.8
Did not respond	15.0	4.2
Educational attainment		
Elementary school	7.0	1.9
High school without diploma	71.0	19.7
High school with diploma	103.0	28.6
Postgraduate education	4.0	1.1
College degree	172.0	47.8
Did not respond	3.0	0.8

### 3.1 Enrollment pathways and recruitment sources

Recruitment for the ProstaPilot study was facilitated through the following channels: Institution Website (16 participants, 3.4%), PR Events (100 participants, 21.4%), Other (140 participants, 30%), External Physicians (20 participants, 4.3%), and Cancer Prevention Center (190 participants, 41%). Recruitment for the ProstaPilot study through “Other” reasons included the following: TV (5 participants, 3.6%), Flyer (1 participant, 0.7%), Friend (68 participants, 48.9%), Employer (34 participants, 24.5%), Family/Relative (26 participants, 18.7%), Institution Employee (8 participants, 5.8%), social media (1 participant, 0.7%), and Congress (1 participant, 0.7%).

#### 3.1.1 Reasons for participation in the ProstaPilot study

At the start of the study, we were interested in understanding the reasons why participants chose to join the study. The Table 3 summarizes the key motivations.

**Table 3:** Reasons for participation in the ProstaPilot study (*N* = 360).

What motivated you to participate in the preventive screening as part of the ProstaPilot project? Select all that apply.	*N*	Percentage (%)
Opportunity for priority access to modern preventive examinations	228	63.5
Motivation to contribute to prostate cancer prevention research	156	43.5
Preventive program/campaign	130	36.2
Recommendation from a friend	80	22.3
Fear of neglecting something important	78	21.7
Fear of being in a high-risk group	62	17.3
Concern for one’s health	50	13.9
Information from the media	48	13.4
Recommendation from a physician	45	12.5
Information from a public figure	44	12.3
Conversation with someone who has been ill or knows someone affected	27	7.5
Recommendation from children	26	7.2
Physical discomfort	13	3.6
Sense of responsibility to the doctor who recommended the examination	11	3.1
Opportunity for priority access to modern preventive examinations (duplicated)	8	2.2
Other	18	

In addition to predefined categories, 18 men provided unique reasons for enrolling in a prostate cancer screening program, highlighting diverse and individual motivations not captured by the main survey categories. The primary motivators identified among the respondent group include: Medical referrals: a significant number of participants joined the program based on referrals from healthcare professionals, including nurses at the institutional prevention unit; Family history: a notable proportion of men were influenced by a family history of prostate cancer, particularly those with affected fathers, underscoring the role of genetic predisposition in health prevention strategies; Family support: some men were encouraged by their family members, especially their wives, highlighting the critical role of familial support in making health-related decisions and Employer incentives: in certain instances, employers provided access to prevention programs as part of their employee benefits, demonstrating a corporate commitment to employee health.

#### 3.1.2 Decision-making timeline for participation in prostate cancer screening

Table 4 summarizes the participants’ responses regarding the decision-making timeline for considering prostate cancer preventive examinations (Table 4).

**Table 4:** Decision timeline for prostate cancer preventive examination participation.

How long did you consider undergoing preventive prostate cancer screening before deciding to participate in the ProstaPilot project?	*N*	Percentage (%)
Immediate (with ProstaPilot)	184	51.1
Days	88	24.4
Weeks	31	8.6
Months	34	9.4
Years	20	5.6
Did Not Respond	3	0.8

A significant majority, 51.1% of respondents (184 out of 357), reported that they had not considered undergoing a preventive examination prior to learning about ProstaPilot, indicating a spur-of-the-moment decision influenced by the program. Others took more time to decide, with 24.4% making up their minds within days, 9.4% within months, and a smaller fraction, 5.6%, pondering over it for years. This distribution suggests that the ProstaPilot program played a critical role in prompting a large portion of participants to take immediate action toward their health.

#### 3.1.3 Perceived impact of phone appointment requirement on study participation

To assess whether the requirement to make a phone appointment posed a barrier to participation, the following item was included in the questionnaire: ‘In your opinion, does the need to make a phone appointment for the study discourage participation?’ A total of 359 respondents evaluated this aspect, assigning an average score of 9.0 (SD = 1.8), where “1” indicated ‘complicates participation’ and “10” indicated ‘no influence on participation’ or ‘would recommend.’

#### 3.1.4 Pros and cons of prostate cancer screening participation

Responses to the 36 items representing the pros and cons of participating in a prostate screening program were analyzed using PCA with Oblimin rotation to account for common variance in the individual barriers and benefits. A parallel analysis identified four reliable factors, explaining 55.6% of the total variance. These are summarized in Table 1.

These factors can be described as follows:

1. Concerns (F1): this factor highlights apprehensions related to the screening process, including fears of unnecessary surgery (loading = 0.899), examinations (loading = 0.789), loss of control (loading = 0.752), and life-altering diagnoses (loading = 0.724). Mean scores for these items ranged from 1.3 to 2.1, with relatively low variability (SD = 0.8–1.2), indicating that these items mostly had no influence or just minor influence on the participants’ decision to participate in the screening program.
2. Benefits (F2): benefits emphasize the perceived advantages of participation, such as the belief that early detection improves cure rates (loading = 0.887), enhances control over one’s health (loading = 0.869), and offers reassurance of being healthy (loading = 0.837). These items achieved high mean scores (3.8–4.5) with moderate variability (SD = 1.2–1.5), reflecting strong agreement on the value of early screening.
3. Social Motivation (F3): social motivation captures the influence of perceived social commitments, including contributing to medical research (loading = 0.610), strengthening relationships with healthcare providers (loading = 0.672), and serving as a role model for others (loading = 0.558). Mean scores for these items ranged from 2.4 to 4.0, with greater variability (SD = 1.4–1.6), suggesting a diverse range of motivational factors among participants.
4. Barriers (F4): barriers describe logistical and social challenges such as time constraints (loading = 0.437), embarrassment during intimate examinations (loading = 0.466), and gender preferences for healthcare providers (loading = 0.778 for “my doctor is a man”). These items had lower mean scores (1.1–1.6) but showed variability, reflecting individualized experiences of inconvenience or discomfort.

#### 3.1.5 Factor correlations

Out of the four factors, Concerns (F1) and Barriers (F4) showed a moderate positive correlation (*r* = 0.48), suggesting that fears about the process tend to coincide with practical obstacles. A weak positive correlation (*r* = 0.27) was also observed between Benefits (F2) and Social motivation (F3). The other correlations were negligible (ranging between −0.11 to 0.05). The weakest relationships were observed between Concerns (F1) and Benefits (F2) (*r* = 0.05) and between Barriers (F4) and Benefits (F2) (*r* = 0.03), indicating that neither worry nor perceived challenges had a significant impact on the participants’ recognition of the screening’s advantages. No significant relationships were observed between the identified factors and participants’ level of education, marital status, or parental status.

#### 3.1.6 Subjective health and its influence on motivation to participate in the study

To examine whether participants’ perception of their subjective health influenced their motivation to join the ProstaPilot study, respondents were asked to rate their current health on a scale from 1 to 10, where “1″ indicated “very poor health” and “10″ indicated “very good health.” Among the 339 respondents, the average self-rated health score was 7.7 (SD = 1.4), suggesting that participants generally perceived their health as fairly good. Responses clustered around scores of 7 and 8, with few individuals rating their health as either poor (1–3) or exceptionally good (9, 10). Importantly, no significant correlations were found between self-rated health and any factors influencing participation in the study or the time taken to decide to participate. This indicates that subjective health perception had limited direct influence on the decision-making process or the motivations for entering the study.

#### 3.1.7 Participant awareness and knowledge regarding on prostate cancer and screening

Understanding public knowledge and awareness of prostate cancer and its prevention is crucial for designing effective health interventions and educational campaigns. This section focused on the levels of awareness and perceptions among participants regarding key aspects of prostate cancer, including its prevalence, risk factors, and the role of screening tools like the PSA test. By exploring these dimensions, the findings offer insights into the knowledge gaps and misconceptions that may influence men’s attitudes toward screening and prevention efforts (Table 5). These insights are vital for tailoring strategies to improve engagement in preventive health behaviors and address barriers to early detection.

**Table 5:** Knowledge-based results on prostate cancer and screening.

Question	*N*	Yes (*N*, %)	Unsure (*N*, %)	No (*N*, %)
Do you agree that prostate cancer is the most common cancer in men?	345	156 (45.2%)	182 (52.8%)	7 (2.0%)
PSA test (Prostate-Specific Antigen) is a blood test that can help detect cancer before symptoms appear. Have you heard about it?	358	259 (72.3%)	33 (9.2%)	66 (18.4%)
The risk of developing prostate cancer increases with age.	357	329 (92.2%)	28 (7.8%)	0 (0.0%)
Preventive prostate cancer screening can be painful and unpleasant.	355	80 (22.5%)	170 (47.9%)	105 (29.6%)
Men over 50, even those without symptoms, should undergo preventive screening.	357	337 (94.4%)	18 (5.0%)	2 (0.6%)

The knowledge survey revealed significant variability in participants’ awareness and perceptions regarding prostate cancer and its prevention. A total of 345 respondents answered whether they agreed with the statement that prostate cancer is the most common malignancy among men. Of these, 45.2% agreed, 52.8% were unsure, and 2.0% disagreed. This highlights a substantial gap in awareness of prostate cancer prevalence even among men with generally higher education who are participating in a volunteer-led screening initiative.

Awareness of the PSA test was more prevalent, with 72.3% of 358 respondents indicating they had heard of it, while 18.4% had not, and 9.2% were uncertain. This suggests relatively high familiarity with PSA as a screening tool within our sample.

Regarding the association between age and prostate cancer risk, 92.2% of 357 respondents correctly recognized that the risk increases with age, and none disagreed with the statement. Similarly, 94.4% of respondents agreed that men over 50, even those without symptoms, should undergo preventive screening, demonstrating strong awareness of the importance of age-related screening.

Participants were divided on the statement that prostate cancer screening can be painful or unpleasant, with 22.5% agreeing, 47.9% unsure, and 29.6% disagreeing. This indicates mixed perceptions, likely influenced by individual experiences or limited knowledge of contemporary screening techniques.

#### 3.1.8 Perceptions of risk and preventive screening

**Figure 1:**
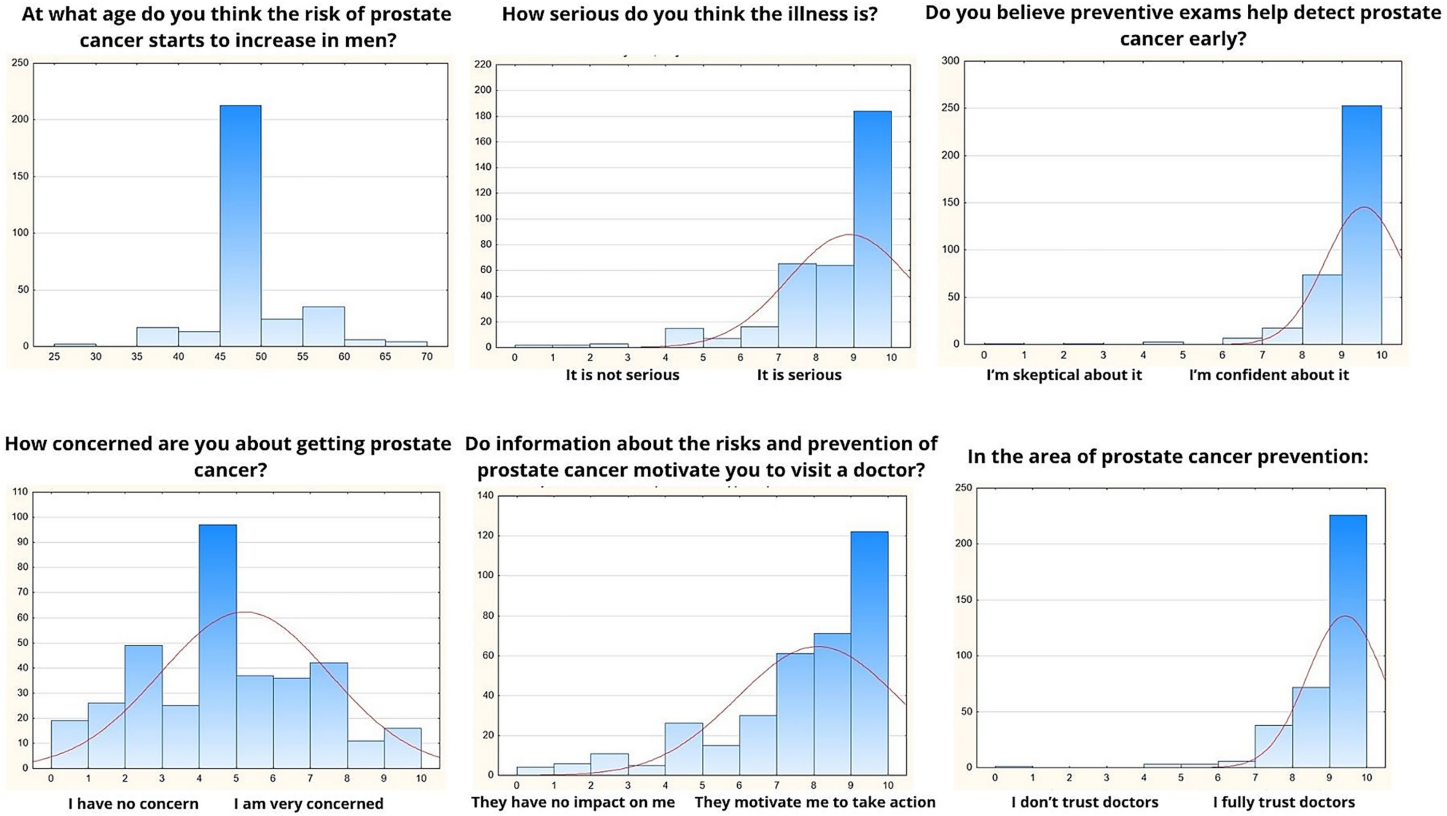
Perceptions of risk and preventive screening.

The questionnaire also contained questions asking the participants how they understood prostate cancer risk, their views on its severity, and the role of preventive measures in motivating health-related actions. Figure 1 illustrates participants’ perceptions of prostate cancer risks, severity, and the effectiveness of preventive measures. Among 346 respondents, the average perceived age at which prostate cancer risk starts to increase is 51.1 years (SD = 5.5), reflecting a broad awareness that aging is a key risk factor. Respondents also rated the seriousness of prostate cancer with a high average score of 8.88 (SD = 1.63), indicating a strong consensus about the gravity of the disease. Confidence in the effectiveness of preventive screenings was equally strong, with a mean score of 9.55 (SD = 0.98), highlighting recognition of early detection as valuable. Participants expressed moderate concern about their personal risk of developing prostate cancer, with an average concern score of 5.23 (SD = 2.29), showing variability in perceived vulnerability. Information about prostate cancer risks and prevention was found to be a significant motivator for seeking medical attention, with an average score of 9.4 (SD = 1.2), underscoring the importance of awareness. Additionally, trust in doctors emerged as a key factor in preventive care decisions, as evidenced by a mean score of 9.6 (SD = 1.0), suggesting high reliance on healthcare professionals for guidance in prostate cancer prevention.

#### 3.1.9 Communication and information sources about prostate cancer prevention

The survey examined two critical aspects of prostate cancer awareness: communication with healthcare professionals (Table 6) and access to information about prevention and risks (Table 7). When asked about their discussions with doctors, the results highlighted significant variability in the depth and impact of these interactions. While nearly half of the respondents reported never discussing preventive screening with a doctor, others indicated conversations that ranged from brief mentions of screening to receiving clear, actionable recommendations.

**Table 6:** Communication with healthcare providers regarding screening.

Have you ever talked to a doctor about preventive prostate cancer screening?	Number (*N*, %)
No	175 (48.6%)
Yes, but only briefly	53 (14.7%)
Yes, the doctor mentioned the option, but no specific recommendations followed (e.g., blood tests or another plan).	42 (11.7%)
Yes, but the doctor’s explanation about the necessity of preventive screening did not convince me.	3 (0.8%)
Yes, and the doctor gave me clear recommendations on what to do.	83 (23.1%)

**Table 7:** Sources of information about prostate cancer prevention and Figure 2.

Source of information	Number (*N*, %)
No	78 (21.7%)
From a doctor	86 (23.9%)
From a friend/colleague at work	68 (18.9%)
From a spouse/partner	52 (14.4%)
From someone in the extended family	26 (7.2%)
From media (TV, social media, newspapers, radio)	164 (45.6%)
Somewhere else	22 (6.1%)
Did not answer	114 (31.7%)

Regarding access to specific information about prostate cancer prevention and risks, the survey revealed that media sources, including TV, social media, newspapers, and radio, played a dominant role in disseminating information, far outpacing personal interactions with doctors or family members. Nevertheless, a substantial number of respondents did not receive any information, and a notable portion did not respond to this question, suggesting potential gaps in outreach efforts. These findings underline the importance of targeted communication strategies to ensure that accurate and actionable information about prostate cancer prevention reaches diverse audiences effectively.

**Figure 2:**
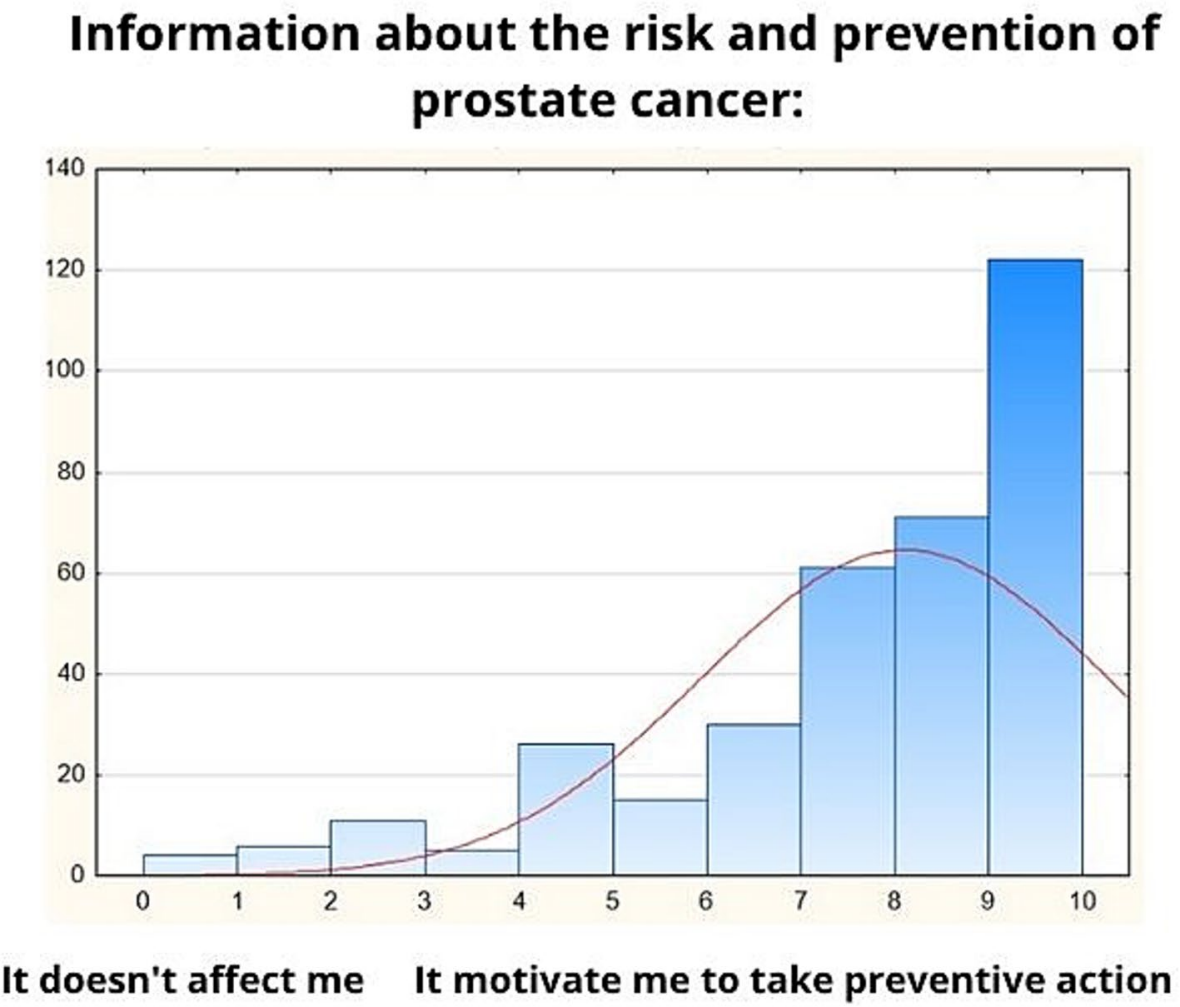
Information about the risk and prevention of prostate cancer.

The Figure 2 demonstrates that information about the risks and prevention of prostate cancer strongly motivates men to take preventive action, as indicated by an average score of 8.11 (SD = 2.17) among 351 respondents. The majority of participants selected responses on the higher end of the scale, particularly 9 and 10, reflecting a high level of motivation. This suggests that well-communicated information about prostate cancer risks and prevention has a significant impact on encouraging men to engage in proactive health measures, such as preventive screenings.

#### 3.1.10 Barriers, beliefs, and social influences in prostate cancer prevention

**Figure 3:**
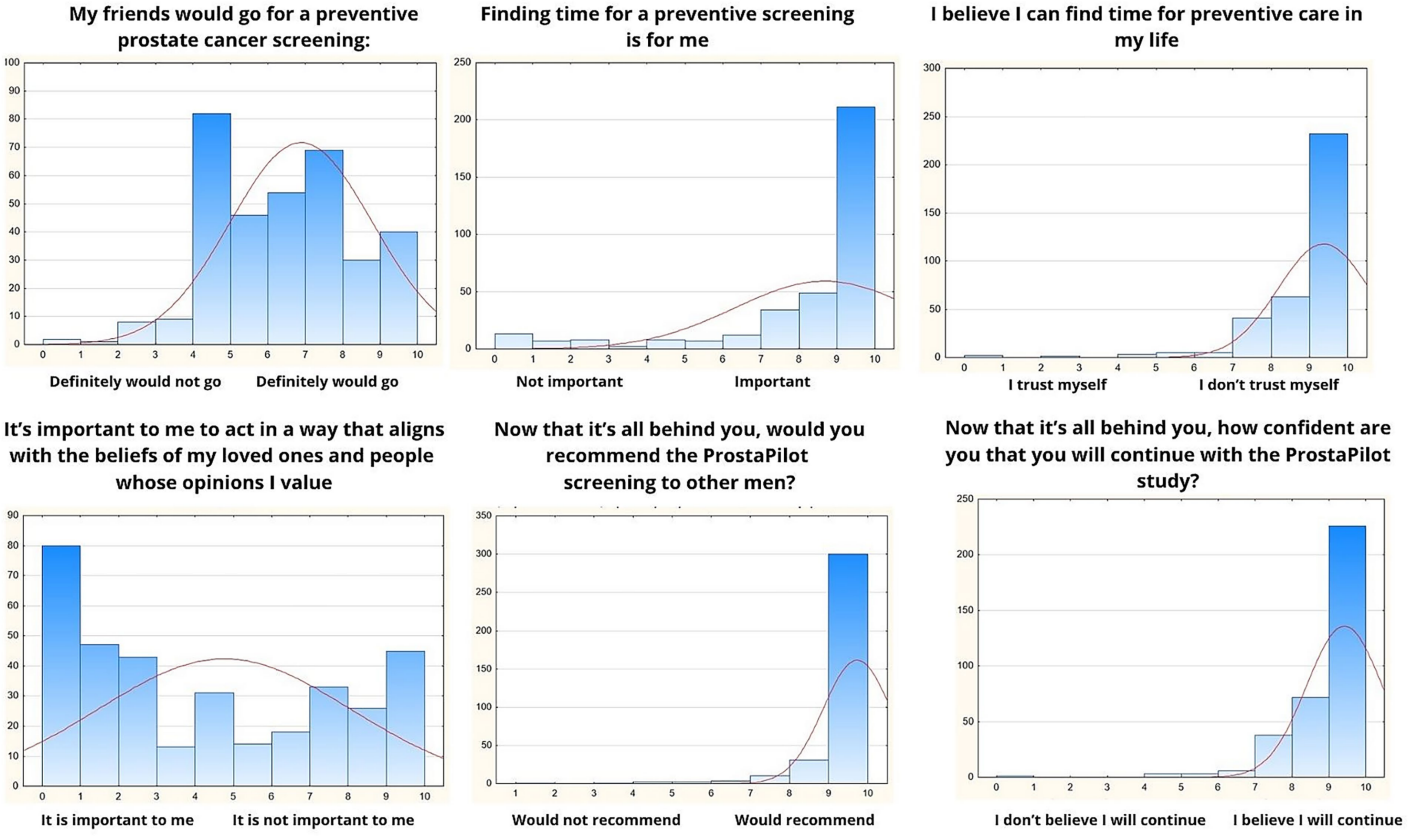
Barriers, beliefs, and social influences.

The final part of the questionnaire focused on understanding the barriers, beliefs, and social influences that shape participants’ decisions to engage in prostate cancer prevention and their commitment to the ProstaPilot program. The responses are summarized in Figure 3. Participants generally agreed that their friends would likely engage in preventive prostate cancer screening, with a moderate mean score of 7.8 (SD = 2.3), indicating some social influence. The ability to find time for preventive care was rated highly, with an average score of 9.73 (SD = 0.87), reflecting minimal perceived time constraints. Similarly, participants expressed strong confidence in their ability to prioritize preventive care in their lives, with a mean score of 9.89 (SD = 0.39). Acting in alignment with the beliefs of loved ones and valued opinions was moderately important to participants, as shown by a mean score of 8.11 (SD = 2.17), highlighting the role of social and familial considerations. After completing the program, participants were highly likely to recommend ProstaPilot screening to others, with a mean score of 9.45 (SD = 1.22), suggesting a positive overall experience. Furthermore, confidence in continuing with the ProstaPilot study was exceptionally high, with an average score of 9.9 (SD = 0.39), underscoring strong participant satisfaction and commitment.

To further assess participants’ attitudes toward prostate cancer prevention, we explored their willingness to recommend screening to others when provided with information about the prevalence and severity of the disease. If participants were informed that prostate cancer is the most common malignancy in men and is often incurable, an overwhelming majority (95.6%, *n* = 344) indicated they would recommend preventive screening to other men. Only 1.9% (*n* = 7) expressed uncertainty, and none of the respondents disagreed. A small percentage (2.5%, *n* = 9) did not answer the question.

#### 3.1.11 Participant perspectives on the study experience

The Table 8 outlines the sources of discomfort and barriers reported by participants during the entire process of the prostate cancer study, from initial appointment to the completion of the MRI examination. Participants rated these challenges on a scale from 1 (no discomfort or barrier) to 5 (significant discomfort or barrier).

**Table 8:** Source of discomfort/barrier.

Source of discomfort/barrier Reflecting on the entire process, from scheduling to undergoing the MRI, the greatest source of discomfort and barrier to participating in and continuing the study was:	*N*	Mean (M)	SD
No influence - score 1…. Has an influence - score of 5			
Filling out questionnaires	347	1.67	0.96
Filling out documents	344	1.59	0.85
Two-day restrictions (e.g., no sex, no cycling) before the examination	341	1.43	0.78
Immediate preparation before MRI (passing gas, bowel movement)	341	1.41	0.83
Need to travel to imaging facility	341	1.39	0.85
Commitment for a long period	337	1.36	0.77
Clarity of information about the study	339	1.33	0.92
Time demands of the examination	342	1.32	0.68
Need to make an appointment by phone with Cancer Prevention Center	336	1.3	0.79
Need to travel to the MOÚ prevention center	342	1.3	0.72
Undergoing the MRI examination	339	1.24	0.67
Communication with staff at Cancer Prevention Center	340	1.22	0.78
Blood sample collection	340	1.18	0.6
Communication with staff during the MRI examination	341	1.14	0.63

The two greatest sources of discomfort identified were filling out questionnaires (*M* = 1.67, SD = 0.96) and documents (*M* = 1.59, SD = 0.85). These were followed by restrictions imposed two days prior to the examination, such as avoiding cycling or sexual activity (*M* = 1.43, SD = 0.78), immediate preparations before the MRI, such as bowel emptying (*M* = 1.41, SD = 0.83), the need to travel to the imaging center where the MRI was (*M* = 1.39, SD = 0.85), and the long-term commitment required for the study (*M* = 1.36, SD = 0.77). As seen in Table 8, ratings for all items were generally low, suggesting only minor source of discomfort.

Items that were rated particularly low as sources of discomfort included blood sample collection (*M* = 1.18, SD = 0.60), communication with the staff during the MRI (*M* = 1.14, SD = 0.63), and communication with the staff at the Cancer Prevention Center (*M* = 1.22, SD = 0.78). These results indicate that while certain procedural and logistical elements posed minor challenges, participants generally found the overall process manageable, with no aspect receiving an average score indicating significant discomfort. This reflects a well-organized study protocol with minimal barriers to participation.

The final set of questions was designed to explore whether, after going through the entire process and understanding what the study entails, participants would recommend the screening and continue advocating for it. These questions were administered after participants completed the study procedure and are presented in Figure 3. The first question explored participants’ willingness to advocate for preventive screening by asking, “If you knew that prostate cancer is the most common malignancy in men and often incurable, would you recommend preventive screening to others?” Among 351 respondents, the average score was 9.73 (SD = 0.87), reflecting an overwhelmingly positive attitude toward recommending preventive screening, with minimal variability in responses. The second question assessed participants’ confidence in preventive measures, asking, “How confident are you in the effectiveness of preventive screening for prostate cancer?” With a mean score of 9.45 (SD = 1.22) from 349 respondents, the results demonstrated a high level of confidence in the effectiveness of screening, underscoring a strong consensus despite slightly greater variability in responses compared to the first question.

## 4 Discussion

The ProstaPilot study has provided valuable insights into the perceptions, attitudes, and behaviors of participants regarding prostate cancer screening. This research enhances our understanding of how highly motivated men view and interact with screening programs. Recent research identifies diverse subgroups of men according to their motivations and barriers to prostate cancer screening (8, 11). This taxonomy is crucial for contextualizing the results of the ProstaPilot study, as it highlights that our participants represent a highly motivated subgroup. This group is distinct from those Ferrante describes, which include “Active refusers,” who are skeptical of the benefits and deterred by misconceptions; “Passive avoiders,” who are open to screening but face logistical or communication barriers; and “Men with abnormal tests,” who are engaged in ongoing screening due to previous abnormal results and require continual support and accurate information (8).

Our sample also differs from the general male population in the Czech Republic, particularly in terms of educational attainment, with a significantly higher proportion of university-educated men included in the study. In the Czech Republic, according to the latest Census data (20), approximately 20.8% of the population aged 15 and over has a university degree. However, in the 50–69 age group, the proportion of men with tertiary education is lower, as the percentage of university-educated individuals generally increases in younger generations. It is estimated that around 10–15% of men in the 50–69 age group hold a university degree. In contrast, our sample includes 47.8% of men with a university education, which is significantly higher than the general population. This difference likely indicates a selection bias, as more educated men tend to participate in health screenings more frequently.

The heightened motivation observed in our study participants may also be attributed to the distinct screening approaches employed, which differ notably from typical methods. Our study’s findings resonate with recent evidence from the IP1-PROSTAGRAM study, which (13) demonstrated a clear preference for MRI over PSA and ultrasound in prostate cancer screening (13). Participants in ProstaPilot program similarly showed high levels of engagement and willingness to recommend the screening program to others, with a notable average recommendation score of 9.45 (SD = 1.22). This aligns with the observed preference for MRI, suggesting that less burdensome and more acceptable screening methods could enhance participation rates and satisfaction. Just as the majority of participants in the IP1-PROSTAGRAM study preferred MRI, reflecting its minimal anxiety, burden, and discomfort (13), our participants also exhibited high confidence in continuing with the ProstaPilot study (mean score of 9.9, SD = 0.39) and a strong social influence indicating their friends would likely engage in similar preventive measures (mean score of 7.8, SD = 2.3).

### 4.1 Pros and cons

The data from the ProstaPilot study indeed suggest that perceived fears and barriers regarding prostate cancer screening only played a minor role in the decision-making of our participants. This interpretation is supported by the low mean scores for concerns (e.g., fear of unnecessary surgery, mean 1.7; fear of losing control, mean 1.6) and barriers (e.g., embarrassment during intimate exams, mean 1.6; time constraints, mean 1.6), which fall significantly below average on the scale used. These results align with findings in the literature that highlight the need to address these issues but also show they may not dominate the decision-making process for many participants (3, 8).

Conversely, the higher mean scores for perceived benefits (e.g., early detection increases the chance of a cure, mean 4.5; assurance of being healthy, mean 4.2) and social motivation factors (e.g., contributing to medical research, mean 4.0) clearly illustrate that these are the key motivators for participation. This is consistent with previous research emphasizing the importance of psychological reassurance, health control, and relational or altruistic motivations in encouraging prostate cancer screening (9, 10).

Our Pros and Cons scale, utilized within the ProstaPilot study to assess participants’ attitudes towards prostate cancer screening, is a bespoke instrument developed from clinical curiosity and previous research. This scale aggregates the pros and cons derived from a thorough review of similar tools. Its development was guided by the Transtheoretical Model of Behavior Change (15), particularly the construct of decisional balance, which evaluates perceived benefits and barriers to behavioral change. The scale incorporates domains such as concerns, benefits, social motivations, and barriers, informed by key studies on prostate cancer screening (3, 8–10). Although its factorial structure is yet to be confirmed in future studies, the scale’s foundation in clinical insights and empirical research positions makes it a potentially valuable tool for scholarly and practical applications.

### 4.2 Trust and information

While trust in healthcare professionals in our study is high (mean score 9.6), the majority of participants did not perceive the information provided by doctors as actionable or sufficient. Our survey results revealed significant variability in patient-physician interactions about prostate cancer prevention. Nearly half of the respondents reported never discussing preventive screening with a doctor, highlighting a substantial communication gap. For those who had discussions, the depth ranged from brief mentions to detailed, actionable recommendations, but only a minority felt fully convinced by their doctor’s explanations. This discrepancy suggests that while doctors hold a position of trust and influence, their communication often lacks depth and specificity, limiting its motivational impact.

Interestingly, our findings also demonstrate that when men receive clear information about the risks and prevention of prostate cancer, it significantly motivates them to take preventive actions (average score of 8.11, SD = 2.17). Moreover, a significant majority, 51.1% of respondents (184 out of 357), reported that they had not considered undergoing a preventive examination prior to learning about ProstaPilot, indicating a spur-of-the-moment decision influenced by the healthcare professional recommendation. This observation is corroborated by Le Bonniec et al. (21), who found that healthcare professionals’ recommendations and the quality of patient-provider communication strongly influence screening participation across various health conditions.

One possible explanation for the lack of strong screening recommendations from physicians lies in their knowledge and beliefs about the efficacy of prostate cancer screening. Bell et al. (22) found that higher knowledge scores among physicians regarding prostate cancer screening were associated with less belief in the mortality benefits of PSA testing (*r* = 0.49, *p* < 0.001). These findings suggest that physicians with higher knowledge may be more cautious about recommending routine PSA screening due to their understanding of its limitations and potential harms. This highlights the need for campaigns that enhance physicians’ understanding of modern screening programs combining PSA testing with the selective use of bpMRI in high-risk men to mitigate the limitations and potential harms of PSA, while also empowering them to engage in effective shared decision-making with patients.

However, contrasting these insights, the systematic review by Riikonen et al. (10) reported no significant impact of decision aids on the frequency of discussions between physicians and patients, nor on men’s decisions to undergo screening. This suggests that while decision aids improve knowledge and reduce decisional conflict, enhancing the quality of conversations between patients and physicians might require additional strategies that go beyond the provision of decision aids alone. To address these challenges, it is crucial to implement strategies that ensure healthcare providers consistently offer clear, detailed, and personalized recommendations about preventive screenings. Training programs should focus on equipping doctors with effective communication techniques and standardized messaging about the importance of early detection. Additionally, incorporating preventive screening discussions into routine consultations could help bridge this gap and make such conversations a norm in clinical practice. Effective communication, as highlighted by Le Bonniec et al. (21), remains a pivotal element in encouraging proactive health behaviors and could be further leveraged by incorporating decision aids into routine patient education to reinforce discussions and ensure that patients receive and understand critical information regarding prostate cancer screening.

Just knowledge is not enough for action.

The findings from Morlando (9) study on prostate cancer screening offer a compelling parallel to our research, demonstrating how knowledge and attitudes significantly influence screening behaviors. Our study revealed that 72.3% of participants were aware of the PSA test, aligning closely with Morlando’s findings where 72.7% were aware of this screening method. Despite this awareness, both studies noted a significant gap between knowledge and actual screening practices: only 29.6% of respondents in Morlando’s study had undergone a PSA test, compared to a slightly higher engagement in our study, suggesting a similar trend of underutilization despite awareness.

Furthermore, both studies emphasize the critical role of effective communication in bridging the gap between knowledge and action. While our research highlighted that 94.4% of respondents believe men over 50 should undergo preventive screening, reflecting a strong recognition of age-related risk, Morlando (9) study also highlighted a robust willingness to screen, with 59.4% expressing intent to undergo the PSA test in the future. These results suggest that while the intention is relatively high, actual screening uptake remains disproportionately low, underscoring the need for targeted interventions to convert positive attitudes into preventive health actions.

In summary, men who volunteered for MRI-based screening were highly motivated, driven chiefly by perceived benefits and social factors, with concerns and practical barriers playing a minor role. Trust in healthcare professionals was high, yet communication about screening was often insufficient. These findings support targeted, socially supportive, clinician-anchored communication to enhance uptake. Generalizability is limited by our motivated sample; future work should test strategies in less-motivated groups.

### 4.3 Limitations of the study

The present study provides valuable insights into the perceptions, attitudes, and behaviors of men regarding prostate cancer screening. However, several limitations should be acknowledged. First, our sample is not representative of the general male population in the Czech Republic because it consists of men who had already decided to participate in the prostate cancer screening programme; this self-selection limits generalizability to less-motivated groups. Second, the cross-sectional design precludes causal inferences about motivational drivers.

Importantly, the high percentage of university-educated men in our cohort (47.8%), compared to the estimated 10–15% of men in the 50–69 age group with tertiary education, should be regarded as a finding with practical implications rather than a limitation. This overrepresentation likely reflects selection patterns whereby more educated individuals are more proactive about health screenings. This finding should inform future action to encourage participation among underrepresented groups (e.g., men with lower educational attainment or lower baseline motivation) through targeted outreach and tailored communication.

Second, the study focuses on highly motivated participants, which limits its generalizability. These individuals may not face the same barriers or possess the same attitudes as other subgroups, such as “Active refusers,” “Passive avoiders,” or those with prior abnormal test results who require ongoing support (8). Further research is needed to explore these underrepresented groups and understand their unique challenges and motivators.

Third, the cross-sectional design of this study restricts our ability to draw causal inferences. For example, while we observed a high level of trust in healthcare professionals (mean score 9.6), the study design does not allow us to determine whether this trust directly influences participation rates or if it is a byproduct of other factors, such as prior positive experiences with the healthcare system.

Additionally, the Pros and Cons scale utilized in this study, while informed by theoretical frameworks like the Transtheoretical Model of Behavior Change (16) is a bespoke instrument and not yet standardized. Future studies should aim to further validate this tool across diverse populations to enhance its reliability and applicability.

### 4.4 Future directions

Future research should focus on exploring underrepresented groups, such as men less motivated to participate in screenings or those facing significant barriers, while employing longitudinal designs to understand trends over time and the effects of interventions. Efforts should aim to improve communication strategies by training healthcare providers to deliver clear, actionable, and personalized recommendations, as well as to standardize and validate tools like the Pros and Cons scale for broader applicability. Investigating alternative, patient-friendly screening approaches, such as MRI, could enhance participation, and targeted educational programs for physicians should address gaps in their knowledge and beliefs about screening guidelines, empowering them to engage in shared decision-making. By addressing these areas, future studies can promote equitable and effective prostate cancer prevention strategies.

## Data Availability

The raw data supporting the conclusions of this article will be made available by the authors, without undue reservation.
